# Supplementary material for: Identification of photoperiod-induced specific miRNAs in the adrenal glands of Sunite sheep (Ovis aries)
Source: Front Vet Sci. 2022 Jul 22;9:888207. doi: 10.3389/fvets.2022.888207 (PMC9354845; doi:10.3389/fvets.2022.888207)
Supplement: Supplementary file 1 [file Data_Sheet_1.ZIP › Supplementary file/miRDeep2 file.zip!/miRDeep2 file/Novel_Structure/LP42A1/Novel_385.pdf]

Provisional ID : NC_019461.2_10124
Score total : 0.9
Score for star read(s) : -1.3
Score for read counts : 0
Score for mfe : 0.6
Score for randfold : 1.6
Score for cons. seed :
Total read count : 16
Mature read count : 16
Loop read count : 0
Star read count : 0

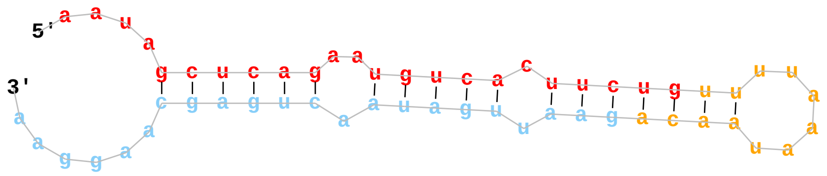

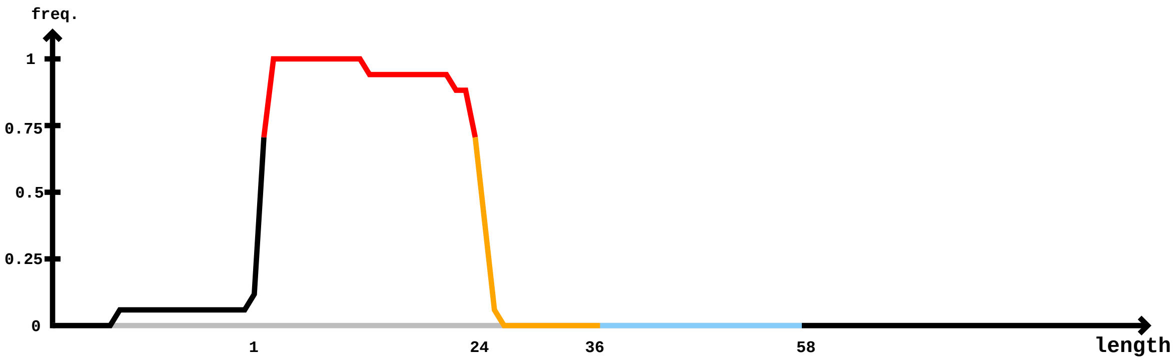

Mature Star

```
5'- uuuguaauucuuuacuguggaauagcucagaaugucacuucuguuuuaaauaacagaauugauaacugagcaaggaaacguaauuuggauuauaaauucuugcuuuaauaaa   -3'    exp
    ((((((((((.((((.((......((((((..(((((.(((((((......))))))).))))).))))))......))))))...))))))))))................   reads      mm         sample
    .....aauucuuuacuguggaauagcucaga.................................................................................   1          0          seq
    ...................gaauagcucagaaugucacuucug.....................................................................   1          0          seq
    ....................aauagcucagaaugucacuu........................................................................   1          0          seq
    ....................aauagcucagaaugucacuucu......................................................................   3          0          seq
    ....................aauagcucagaaugucacuucug.....................................................................   6          0          seq
    .....................auagcucagaaugucacuucug.....................................................................   3          0          seq
    .....................auagcucagaaugucacuucugu....................................................................   1          0          seq
    .....................auagcucagaaugucacuucuguu...................................................................   1          0          seq
```
